# Supplementary material for: A systematic assessment of current genome-scale metabolic reconstruction tools
Source: Genome Biol. 2019 Aug 7;20:158. doi: 10.1186/s13059-019-1769-1 (PMC6685185; doi:10.1186/s13059-019-1769-1)
Supplement: Supplementary file 2 — Review history. (DOCX 2969 kb) [file 13059_2019_1769_MOESM2_ESM.docx]

Review History

**Comments to author and authors’ point-by-point responses to the reviewers’ comments:**

**Reviewer 1**

I want to commend the authors on the hard work that went into this comprehensive benchmarking study of a large number of metabolic model reconstruction tools. I am sure that it will be tremendously helpful to many people that need to make an informed decision about which tool to choose in which context (I'd have loved to have this information available 2-3 years back ...). I have only one larger concern that I think the authors should at least try to address (see below) and a number of smaller comments. The manuscript is well written and comprehensive. Sufficient supplementary information and analysis code (wherever possible) has been provided.

Major revisions
---------------
I think the results from the model content comparisons (set analysis of reactions, genes and metabolites) don't let the different tools look particular good. My gut feeling tells me that the trade-off between coverage vs. additional content (that might or might not be correct; very likely the latter) looks quite bad actually.

*Respons: It is true that the results from the model comparison does not let the different tools look particularly good. We were also quite surprised by the low coverage of the draft networks when we got our first results several months ago. That’s why we looked at the details of our network comparison and we found both genuine and mapping-related reasons for the low coverage.*

*To solve the mapping-related reasons, we focused our efforts on improving the mapping of metabolites in the manually-curated models to known databases. Figures below show the improvement in the mapping of metabolites for the manually curated models of L. plantarum and B. pertussis, respectively. In both cases, the manually-curated models are better represented in the BiGG database. We manually checked all the metabolites which are in the BiGG database and not in the others and we found that these compounds really don’t exist in other databases.*

*Figure 1. Mapping of metabolites in the manually-curated model of L. plantarum to known databases. Bars to the left were calculated using the original model. Bars in the middle were calculated using the model presented in the manuscript revised by the referees. Bars to the right a were calculated with the model presented in the current version of the manuscript*

*Figure 2. Mapping of metabolites in the manually-curated model of B. pertussis to known databases. Bars to the left were calculated using the original model. Bars in the middle were calculated using the model presented in the manuscript revised by the referees. Bars to the right a were calculated with the model presented in the current version of the manuscript*

*As stated in our paper, we also found duplicated metabolites in the BiGG database that were preventing an appropriate mapping with other databases (KEGG, MetaCyc, SEED). For example, 2ttdeacp (*[*http://bigg.ucsd.edu/universal/metabolites/2ttdeacp*](http://bigg.ucsd.edu/universal/metabolites/2ttdeacp)*) is the same as tmrs2eACP (*[*http://bigg.ucsd.edu/universal/metabolites/tmrs2eACP*](http://bigg.ucsd.edu/universal/metabolites/tmrs2eACP)*). However, the first one does not contain links to external databases and the second does. Probably, when the model for L. lactis (iNF517) was submitted to the BiGG database, the authors of that model didn’t map metabolites to the BiGG database properly, and the BiGG database ended with duplicated metabolites.*

*As the manually-curated models had a lot of duplicated BiGG metabolites without links to external databases (like 2ttdeacp), those metabolites couldn’t be mapped to other databases. We therefore manually fixed these metabolites in the manually-curated models using identifiers with links to other databases. To keep the analysis consistent, we also fixed iNF517, but that change implied that we had to run AuReMe and MetaDraft again.*

*Despite the additional improvement in the mapping, we didn’t see a substantial increase in the coverage. Subsequently, we examined the reasons for the low coverage of the tools. Although the main reasons were explained in the manuscript, we may have failed in communicating all of them. As we think it is quite important for the readers to understand them, we expanded some sections in the manuscript and we created new supplementary files (S3-S6 Figures) to give more details to the users.*

*Here we summarize the main reasons for the general bad performance of the tools.*

1. *First,* *both manually-curated models contain a considerable amount of reactions without gene-associations, including spontaneous, transport, exchange reactions, reactions added during the manual gap-filling and biomass-related reactions. For L. plantarum and B. pertussis, there are 241 and 657 of such reactions, representing 31% and 39% of the network, respectively. With the exception of CarveMe and ModelSEED, which can perform automatic gap-filling, all the rest of the tools is not able to recover most of the non-gene associated reactions, mainly because all the tools predict reactions based on genomic evidence. Thus, for both species, around, 50% of the reactions that were not recovered don’t have gene-reaction associations in the manually-curated model. Without considering exchange reactions, the coverage roughly increased by 10% except for CarveMe and ModelSEED.*
2. *Second, in around 30% of the reactions that were not recovered there is at least 50% of the associated genes missing in the draft reconstructions.*
3. *Third, even when all the genes associated to a particular reaction are recovered, specific substrate and cofactor usage is difficult to predict. Many times, the tools predict the correct metabolic activity but they fail in predicting the specific substrate used in the manually-curated models. We created a collection of plain text files containing hundreds of examples where the associated genes were recovered by the tool but the reaction does not correspond to the one in the manually-curated model because of different substrates (see section availability of data for details).*

The problem I have with this analysis is that I don't know exactly how bad the results are without a baseline. Given that manually reconstructed models are considered the gold standard in this benchmarking study, I think the authors should try to establish this baseline by running manually reconstructed networks through their pipeline for a small number of organisms (or 2-3) that have been independently reconstructed by different research groups. It is unlikely that there will be a perfect separation between models, but even a rough estimate of the different metrics would already be useful in evaluating the results for the automatically reconstructed networks. A starting point could be the three independent P. putida models discussed in [1] for example.

*We may start saying that running a species through our pipeline is very time-consuming. In particular, to generate draft models for 1 species with all the tools takes approximately 1 month, mainly because some tools like Merlin are very slow (each run could take up to 200 hours) and the runs in others cannot be automated for a regular user (AuReMe, MetaDraft, Pathway Tools, ModelSEED). In addition, the manually-curated model used as a gold standard have to be mapped to known databases before running the pipeline (otherwise we cannot compare the draft models with the manually-curated ones). Because we chose non-BiGG models, these models are not fully mapped to known databases. The manual revision and mapping of the metabolites to known databases takes at least 1 week per model. The identification of metabolite synonyms which are not in MetaNetX also takes 1 week. The analysis, interpretation and writing of results takes another week. So, to summarize, to run an additional species through our pipeline takes no less than 6 weeks and could take up to 2 months.*

*Second, we trust the quality of our models since we built them ourselves. We know that they accurately predict experimental data (auxotrophies, growth on specific media conditions) and that they don’t have artifacts (for example, ATP production without exchange reactions). We believe that manual curation is not sufficient for a model to be consider a gold standard for this benchmarking study; the model also has to be of high-quality, i.e., predict experimental data in a realistic way. We don’t know the quality of the models suggested by the reviewer but according to the reference, one of the P. putida models is able to produce ATP without nutrients, so we discard that model for this benchmarking study. The other two P. putida models were created by the same group and since one is just the extension of the other, the overlap between them is big. According to the reference, the models can reproduce experimental data accurately, so they can be considered as gold standard. Since one model, is just the extension of the other, we believe that it is enough to run the best (the most updated) of those two models through our pipeline.*

*Consequently, we built 27 draft models for P. putida with all the tools and we compared them with iJP612. The results confirmed our previous findings.*

Minor revisions
---------------
- Maybe the authors could provide a visual decision tree for readers to pick a suitable tool? For example, "Should the reconstruction be simulatable -> Yes or No, ..."

*We thought about that before submitting the article. We did some exercises and concluded that the problem is so complex (too many variables), that it could be misleading for the readers to create a tree with just a few decision variables. A decision tree with 6 questions could miss a lot of important (deal-breaking) factors for potential users. We believe that the decision is in the details and that users have different priorities, so we can’t choose the 6 most relevant features for them. We believe that the users should study the table of features and decide for themselves which one(s) is(are) the more convenient tool(s).*

*Below we describe some examples of decision trees and their corresponding erroneous conclusions.*

*Example decision tree 1:*

*Maybe the user would like to build a model for a eukaryote. He would like to use a tool that generates a working model, with BiGG identifiers. He would conclude that CarveMe is the option but CarveMe does not support eukaryotes. He probably would have to go for Merlin or Pathway Tools if there is no close species, or MetaDraft or AuReMe if there is. Unfortunately, either way, he won’t get a working model.*

*Example decision tree 2:*

*This is a real case: maybe the user wants to build models for several strains integrating data from Metacyc and KEGG. He would conclude that RAVEN is the correct option. However, he works in a company and they cannot pay the license for MATLAB, so RAVEN is not an option.*

*Example decision tree 3:*

*Peter is a biologist with not much background in computer science. He needs a software platform where he can perform the manual curation of a microorganism with no phylogenetically close species. As he likes to use the latest standards, he chose Merlin. However, he also needs to visualize pathways and ideally the whole network. If he would have looked at the table, he would have chosen Pathway Tools because it has much better visualization tools, and maybe he accepts that this tool does not work with the latest standards because that wasn’t so critical for him.*

- Is there a manuscript for metadraft in the works? Can it be shared with the reviewers?

*There is no manuscript for MetaDraft. The software can be publicly accessible through GitHub and a user manual can be found at the same repository. Many people from outside our lab use it and that’s why we decided to include MetaDraft, even though there is no manuscript. There will be a manuscript at the end of the year.*

- While I fully agree that using models from BiGG for the benchmarks would have given some tools (e.g. CarveMe) an unfair advantage, but I am also a little bit concerned that both iLP728 and iBP1870 are models that have been manually reconstructed in the authors' group. How much of an advantage does the use of these models provide to MetaDraft, which has been developed by the group as well?

*That’s a fair question. Both iLP728 and iBP1870 were built independently from MetaDraft. iLP728 was built in 2005 (published in 2006) when there were only a few genome-scale models (E. coli and a few others). For its reconstruction, Pathway Tools and Simpheny (an outdated software tool licenced from Genomatica at the time) were used.*

*iBP1870 was built more recently in 2016 (published in 2017). This model was reconstructed using orthology with iAF1260, a genome-scale model of E. coli*

*In both cases, a massive manual curation was performed so both models cannot be replicated through automatic methods.*

*In conclusion, both models are not connected in any way to MetaDraft. Indeed, the MetaDraft project started in 2016 and the first release was done in this year.*

*To end our comment about the potential bias, we may say that any tool that uses the BiGG database as reference, will perform better than others. In theory, RAVEN is also capable of using the BiGG models, but we obtained an error when using them. We attach the error message we got in the supplementary files.*

- Why was `R` defined as `|in_model| / |additional|` and not as `|in_model| / (|additional| + |not_in_model|)`?

*Because when we looked at figure 5 or 6 and we wondered how similar each draft was to the manually curated model, we thought that a similar draft network would be one with large blue bars (in model) and small yellow bars (additional). Therefore, we just took that ratio as a measurement. We think that this metric is very intuitive for the reader, which would make its interpretation a little bit easier. In addition, we see that there is a dependency between |in_model| and |not_in_model| since in_model + not_in_model = n_rxns, so the ratio suggested could be written as `|in_model| / (|additional| + n_rxns - |in_model|). In the end, it will depend only on |in_model| and |additional|.*

- A little bit surprising that carveme adds so many reactions without GPRs given that formulation actually punishes the use of them. Is it because BiGG as universal reaction database is too small?

*Around 23% and 35% of the reactions without GPRs in the models created with CarveMe are transport reactions and exchange reactions, respectively. Although CarveMe punishes the addition of reactions without GPRs, these kinds of reactions are needed to generate biomass.*

*CarveMe actually doesn’t use the entire BiGG database (24.000 reactions) as a reference database for the gap-filling procedure. CarveMe takes reactions from the template model (around 4300 BiGG reactions) to gap-fill the draft model. Although this is the same approach used by ModelSEED, the template models of ModelSEED have around 8600 reactions. In comparison with ModelSEED, the templates used by CarveMe are indeed smaller. In comparison with ModelSEED, I would expect that because of the smaller template models, less reactions are found during the homology detection step, and therefore more reactions are needed to gap-fill the network.*

- p. 13, l. 227, why are the different numbers of models for L. plantarum and B. pertussis?
*The difference in the number of runs differ for the two species because we couldn’t generate two networks with AuReMe for B. pertussis. We changed the file with the details of the runs that we made. Now, we also specify which runs didn’t work.*

- p. 14, l. 255, totally -> maximally?

*In order to provide a clearer message to the readers, we changed the sentence from “… a value of 1 means that the networks are totally different” to “… a value of 1 means that the networks do not share any element”*

- p. 15, l. 289, important -> large?
*Yes. In that line, we changed the word to “large”. We also changed the word important in other part of the text.*

- p. 18, l. 344, are there plans to provide this information to the metanetx team?

*We contacted the support team of MetaNetX and they told us that it is possible to submit new synonyms. We are going to send the list to the MetaNetX team for revision.*

- p. 18, l. 352, why is the coverage much lower in B. pertussis?

*It is because, in contrast to the model for L. plantarum which only have 2 compartments (cytosol and extracellular space), the model for B. pertussis has 3 compartments (cytosol, periplasm and extracellular space). The draft models of Merlin, Pathway Tools and RAVEN (KEGG) do not include transport and exchange reactions; they include only reactions in the cytosol. Therefore, the large number of reactions in the model of B. pertussis involving the periplasm and extracellular space (824, which represents 49% of the network) are missing in the draft models made with these tools. For plantarum, the number of reactions involving the extracellular space is not so big (234, which represents 30% of the network) which makes easier for these tools to predict the correct reactions.*

- p. 21, l. 435, I thought exchanges reactions are excluded are not considered (p. 430)?
*The reference to exchange reactions in line 435 it is just an example of reactions that never have genomic evidence (they are never associated to genes because they are there just for modeling purposes, i.e., to represent exchange with the environment or media). We removed that example to avoid misunderstandings with line 430.*

*In line 430 we are talking about the manually-curated models and in line 435 about the draft models created with AuReMe and RAVEN.*

References
----------
Yuan, Q., Huang, T., Li, P., Hao, T., Li, F., Ma, H., Wang, Z., Zhao, X., Chen, T. & Goryanin, I. Pathway-Consensus Approach to Metabolic Network Reconstruction for Pseudomonas putida KT2440 by Systematic Comparison of Published Models. PLoS One 12, e0169437 (2017).

**Reviewer 2**

The authors provide an important service to the metabolic modeling community with their comprehensive analysis. They compare seven popular tools for reconstructing genome-scale models, with both qualitative and quantitative metrics. This work is relevant to the readers of Genome Biology. 

There are a couple things that should be addressed before publication:

1. I compared this paper to a similar review from a few years ago: Hamilton JJ, Reed JL. Software platforms to facilitate reconstructing genome-scale metabolic networks. Environ Microbiol. 2014;16: 49-59. doi:10.1111/1462-2920.12312

This is a nice update to the results from that review, and it includes the newer tools. However, a number of the features described by Hamilton & Reed are not assessed here, particularly those used in Manual Curation / Refinement and Network Evaluation (Stages 2 & 4 in that paper).

The difference looks to be one of scope. This manuscript focuses mostly on the Draft Reconstruction process, with a few assessments of curation and refinement (a few rows in Figure 1), but not as much as a potential users might like to know (e.g. can these tools be used for managing confidence scores and notes?). Either this part of the analysis should be expanded, or some additional discussion could be added with reference to the previous article.

*As noted by the reviewer we do have a difference in scope with the mentioned article. In that article, I would say that the question was what I can do with each tool? Here, we are mainly trying to answer in this manuscript how much work would it be to go from the draft network to the final network using each tool.*

*However, we agree with the reviewer in that potential users might like to know specific features during the refinement process. As our main drive is to give a useful assessment for users, we took 18 relevant features related to manual curation from Table 1 in the referenced article, an we assessed those features for each of the tools (supplementary files S25-S26).*

2. The tools were only quantitatively assessed with prokaryotic genomes and metabolic networks. Therefore, the authors cannot give a realistic assessment of the quality of these tools for working with eukaryotes. Figure 1 does mention the ability to reconstruct eukaryotic metabolic networks, but this might be misleading to readers; eukaryotes put many additional demands on the reconstruction process (e.g. compartmentalization), and a high score in the box in Figure 1 isn't a guarantee that the tool will work well in a real application. I recommend adding some discussion on this, and assessment of these tools for eukaryotic reconstructions could be addressed in a future publication.

*The reviewer is correct. Among the new features that we assessed; we now consider “determination of reaction localization”. Some of the tools perform better than others in determining compartments, so now the users can see the details. Maybe some of them will realize that for a particular tool, even though it is able to build models of eukaryotes, the potential difficulties in defining compartments will make it hard to use.*

3. Did this authors consider MetExplore? <http://www.metexplore.fr/>

*No. We included two additional tools in our review: MetExplore and rBioNet. MetExplore is not meant to build a model from its genome but instead to curate a model and rBioNet is a tool to assemble models but it cannot generate a draft from its genome, so we just included these tools in the review section and we did not generate networks with these tools.*

4. L486 "later" should be "latter"

*We fixed it. Thanks.*

5. Should the the axes in Figures 2 and 7 should be labeled JD_g / R_g and JD_r/ R_r, respectively? It might be nice to combine these figures together so they can be compared more easily.
*Yes. Thanks*
